# Marker genes for circulating tumour cells predict survival in metastasized breast cancer patients

**DOI:** 10.1038/sj.bjc.6600868

**Published:** 2003-04-01

**Authors:** B Weigelt, A J Bosma, A A M Hart, S Rodenhuis, L J van 't Veer

**Affiliations:** 1 Division of Experimental Therapy, The Netherlands Cancer Institute, Plesmanlaan 121, 1066 CX Amsterdam, The Netherlands; 2 Department of Radiotherapy, The Netherlands Cancer Institute, Plesmanlaan 121, 1066 CX Amsterdam, The Netherlands; 3 Department of Medical Oncology, The Netherlands Cancer Institute, Plesmanlaan 121, 1066 CX Amsterdam, The Netherlands; 4 Department of Pathology, The Netherlands Cancer Institute, Plesmanlaan 121, 1066 CX Amsterdam, The Netherlands

**Keywords:** breast cancer, marker genes, real-time PCR, prediction

## Abstract

We investigated the prognostic significance of circulating breast cancer cells in peripheral blood detected by quantitative RT–PCR of marker genes in patients with advanced breast cancer. Blood samples from 94 breast cancer patients with metastatic disease (M1) were examined for circulating tumour cells by studying the mRNA expression of CK19, p1B, PS2 and EGP2 by real-time PCR. Using a score function, developed for predicting circulating tumour cells by quadratic discriminant analysis (QDA), the four expression levels were combined into a single discriminant value. Tumour cells were present in 24 out of 94 (31%) of the patients. In 77% (72 out of 94) of the patients distant metastatic disease was localised in the bone. In 36% (26 out of 72) of the patients with bone metastases at the time of blood sampling, a positive QDA for the four genes was found, in contrast to only 14% (three out of 22) without bone involvement. Overall survival rates by Kaplan–Meier revealed no prognostic effect for the presence of bone metastases (*P*=0.93). However, patients with a positive QDA value did have a progression-free survival at 1 year of 3% and overall survival at 2 years of 17%, against 22 and 36% for patients with a negative QDA value (*P*=0.015 and 0.0053, respectively). Breast cancer patients with metastatic disease have a significantly worse progression-free and overall survival when circulating tumour cells can be detected in their peripheral blood.

Metastases in breast cancer may arise through either lymphatic spread or through hematogenous dissemination. While the local treatment of breast cancer is quite successful and modern treatment can adequately deal with local lymph node metastases, a substantial proportion of breast cancer patients ultimately die from metastases to distant organs or tissues. Micrometastatic disease may be cured by adjuvant systemic therapy and the ability to detect it reliably would thus be a significant advance. We have recently shown that the tendency for hematogenous spread can already be shown in small tumours, using messenger-RNA (mRNA) expression profiling (van 't Veer *et al*, 2002). In addition, we and others have developed immunological and RNA-based methods to detect circulating tumour cells in blood, bone marrow and other nonbreast tissues (Lambrechts *et al*, 1998). There is evidence that the presence of epithelial cells in the bone marrow of patients with early breast cancer correlates with prognosis (Braun *et al*, 2000). Antibody-based methods to detect these occult tumour cells have, however, not gained widespread clinical use because of significant numbers of false-positive results (Lambrechts *et al*, 1998,^1999^).

RNA-based methods have been used to detect the presence of mRNA species in RNA isolations from peripheral blood mononuclear cells that are characteristic of epithelial cells and that may originate from circulating tumour cells. The main problem of RNA-based assays continues to be the almost universally present background signal (Traweek *et al*, 1993; Datta *et al*, 1994; Burchill *et al*, 1995; Krismann *et al*, 1995; Fields *et al*, 1996; Moscinski *et al*, 1996; Mapara *et al*, 1997; Zippelius *et al*, 1997). We have recently developed a method that overcomes these problems by making use of two relatively recent developments. First, a truly quantitative PCR has become available, which is known as ‘real-time PCR’ (Bieche *et al*, 1999). Second, we have selected mRNA species using SAGE that are not present in peripheral blood RNA isolates or in bone marrow cells of healthy volunteers, but that are highly expressed in most breast cancers (Bosma *et al*, 2002). A panel of four marker genes (p1B, PS2, CK19 and EGP2) was used to detect circulating tumour cells in the peripheral blood of breast cancer patients; peripheral blood samples of healthy females were used as controls. Using a quadratic discriminant analysis (QDA), a score function based on the mRNA levels of the four genes was derived which, when positive, predicts the presence of circulating tumour cells (Bosma *et al*, 2002). This assay yields positive results in roughly 30% of the patients receiving treatment for advanced breast cancer. Here we show that a positive discriminant value is more frequent in patients with bone metastases and is furthermore associated with a reduced survival.

## MATERIAL AND METHODS

### Patients

This study is based on the same patient series as reported earlier (Bosma *et al*, 2002). One hundred and three patients with advanced breast cancer, who were under treatment at the outpatient clinic of the Netherlands Cancer Institute, were asked to participate in the study. No other selection criteria were applied. Patients receiving hormonal treatment, intravenous administration of bisphosphanates or who were undergoing chemotherapy (or any combination) were eligible. Blood samples were collected during a routine follow-up visit in the Netherlands Cancer Institute/Antoni van Leeuwenhoek hospital between 1997 and 1998. A written informed consent was obtained from all the patients and the study was approved by the institute's protocol review committee.

Patient characteristics and follow-up information were extracted from the clinical charts. These included demographical data, the date of diagnosis of breast cancer, performance status, sites of metastatic disease, haemoglobin, white blood cell (WBC) count, platelets, LDH, CEA and CA15.3. In addition, the presence of either progressive or responding disease at the time of blood sampling was noted. The dates for first treatment progression and death were also extracted from the charts.

For this study, nine of the 103 patients had to be excluded because they were not under further treatment at the NKI and had been lost in follow-up, leaving 94 patients for the analysis.

### Statistics

The QDA score function as defined earlier (Bosma *et al*, 2002) was calculated from the expression levels of the four marker genes p1B, PS2, CK19 and EGP2. A threshold was set that gave the maximum number of correctly classified patients in the breast cancer group (sensitivity) at zero misclassified normal controls (specificity was set to 100%) (Bosma *et al*, 2002).

Survival was calculated from the date of blood sample to the date of last follow-up (censored) or date of death from any cause (event). Progression-free survival was calculated from the date of blood sampling to the date of last follow-up (censored) or date of progression/death from any cause (event). Patients, in whom evidence of progression was present at the date of blood sample, were assigned a progression-free survival of zero months. (Progression-free) survival curves were calculated using the method of Kaplan–Meier and compared using the likelihood ratio test based on proportional hazard regression analysis.

Proportional-hazard regression analysis was used to adjust the association between QDA and survival or progression-free survival for confounding by any other metastastic site (liver, bone, lung or other sites) or form of treatment (chemotherapy, hormonal therapy or both).

The median follow-up from the date of blood sampling for all the 94 patients included is 15 months (range 0.4 – 53) and for the 17 patients still alive 45 months (12 – 53).

### RESULTS

Ninety-four breast cancer patients with metastatic disease were studied for the prognostic significance of positive breast cancer marker gene expression in their peripheral blood. All the patients had metastatic disease, most of them had metastases at several locations. Twelve patients did not receive any systemic treatment at the time of blood sampling, 36 patients received chemotherapy, 39 hormonal treatment and seven received both (patient characteristics, see Table 1

**Table 1:** Clinical characteristics of 94 breast cancer patients with M1 disease

	**No. of patients (*n*=94)**	
**Characteristic**	**No.**	**%**
*Metastases*^a^		
Bone metastasis	72	77
Liver metastasis	27	29
Metastases at other sites	67	71
		
*Systemic treatment*^a^		
Chemotherapy	36	38
Hormonal treatment	39	42
Chemo- and hormonal therapy	7	7
No treatment	12	13
		
Progressive disease^a^	31	33
		
Age at initial diagnosis (year)	48.7	(28 – 80)
Age at blood sampling (year)	54.1	(31 – 82)
		
Interval initial diagnosis–blood (year)	5.4	(0.03 – 21.6)

a At the time of blood sampling.

). The circulating breast tumour cell assay by real-time PCR quantification for the four marker genes p1B, PS2, CK19 and EGP2 revealed that 29 out of 94 patients had a positive discriminant value for circulating breast tumour cells, as reported earlier (Bosma *et al*, 2002).

**Figure 1:**
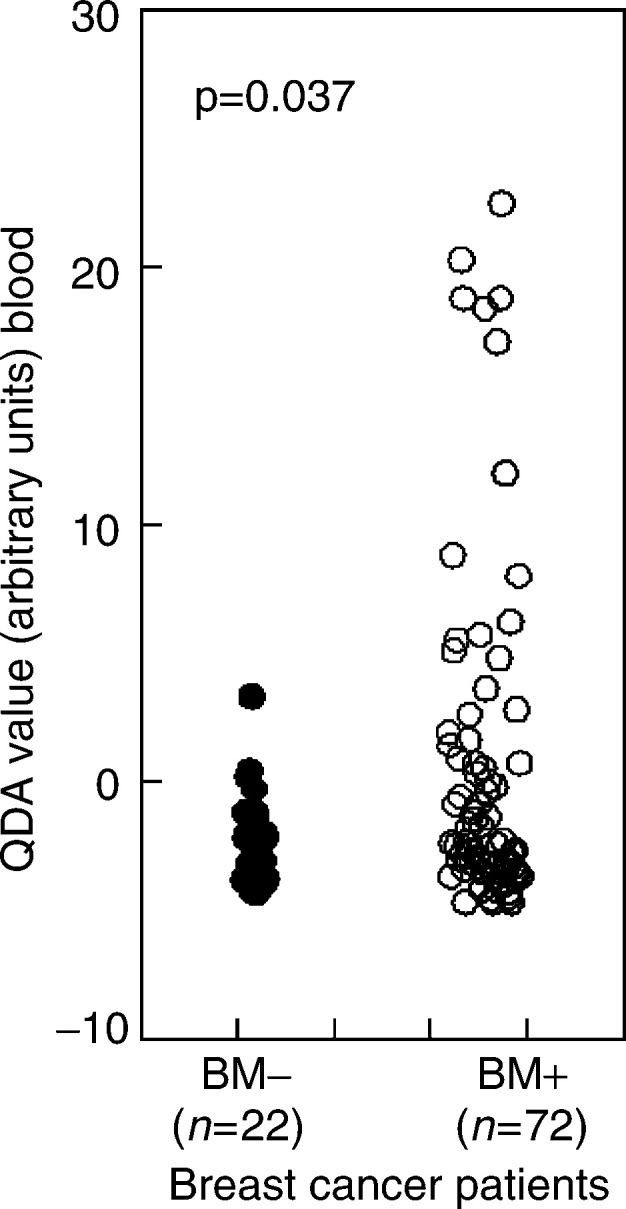
Twenty-six of the 72 patients with bone metastases (BM+) had a positive QDA value against three of the 22 patients without bone involvement (BM−) (*χ*^2^ test *P*=0.037). We previously defined a cutoff value of zero QDA arbitrary units on the QDA score to set the specificity to 100% (Bosma *et al*, 2002).

A positive test for circulating tumour cells was (almost exclusively) found in patients with bone metastases (Figure 1

). Twenty-six of the 72 patients (36%) with bone metastases had a positive QDA value, versus three of the 22 patients (13.6%) without bone metastases (*χ*^2^ test *P*=0.037). A positive QDA value was also associated with liver metastases. Thirteen of the 27 patients (48%) with liver metastases had a positive QDA value, versus 16 of the 67 patients (24%) without liver metastases (*χ*^2^ test *P*=0.022, data not shown). Logistic regression analysis revealed that these associations are independent of each other. The results of the circulating breast cancer cell assay were further correlated with each of the quantitative data extracted from the patient charts. There was no apparent relation with age, the time since primary diagnosis, levels of LDH or tumour markers, WBC counts or platelet count or with the presence of disease progression at the time of sampling.

**Figure 2:**
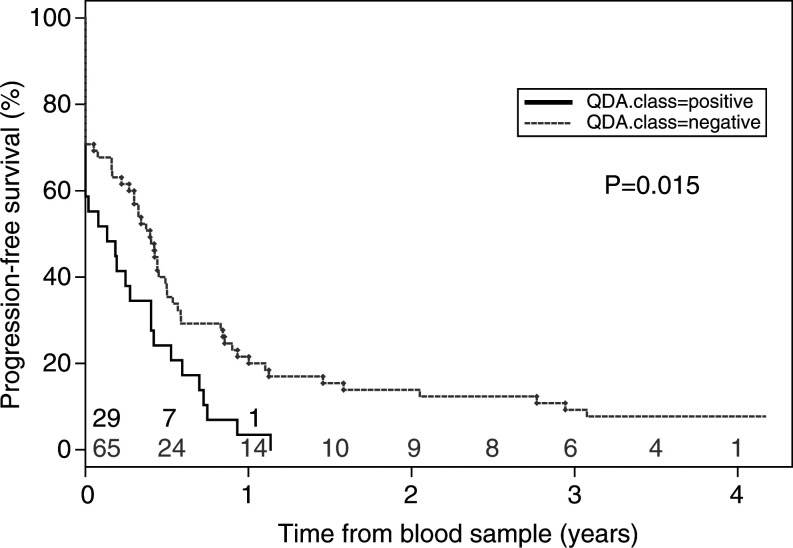
Kaplan–Meier curves for progression-free survival in patients with or without a positive test for circulating tumour cells (likelihood ratio test; *P*=0.015). Patients at risk at each time point (years) are indicated for ‘QDA positive’ (black) and ‘QDA negative’ (grey) patients.

**Figure 3:**
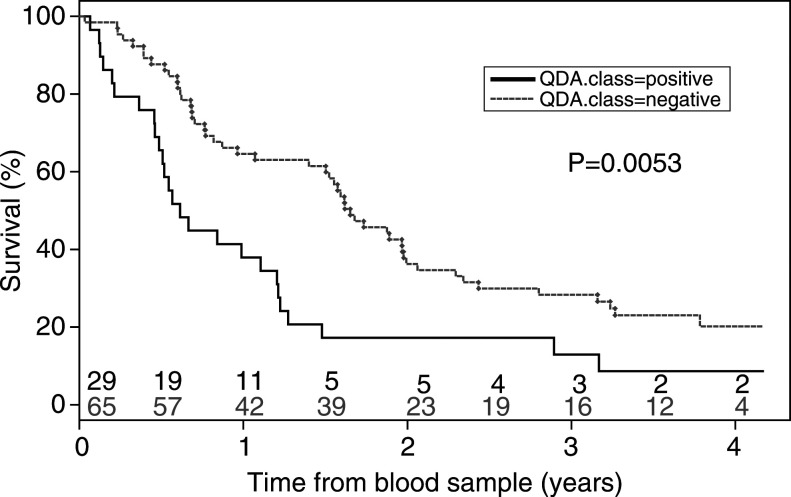
Kaplan–Meier curves for overall survival of patients with or without circulating tumour cells (likelihood ratio test; *P*=0.0053). Patients at risk at each time point (years) are indicated for ‘QDA positive’ (black) and ‘QDA negative’ (grey) patients.

Thirty-three percent of the patients had progressive disease at the date of the blood sampling (31 out of 94). After 6 months, 33% were still alive without progression, after 12 months 16%, after 2 years 10% and after 4 years 5%. There is evidence (*P*=0.015) that progression-free survival is worse for patients with a positive QDA value from the four marker genes (Figure 2

). The presence of circulating tumour cell mRNA in the peripheral blood is clearly associated with a worse overall survival (*P*=0.0053, Figure 3

). The median survival of patients with a positive test was 6 months, while the median survival of patients with a negative test was 18 months. No difference between positive and negative QDA patients was found regarding the interval between diagnosis and blood drawing (*t*-test: *P*=0.71).

**Figure 4:**
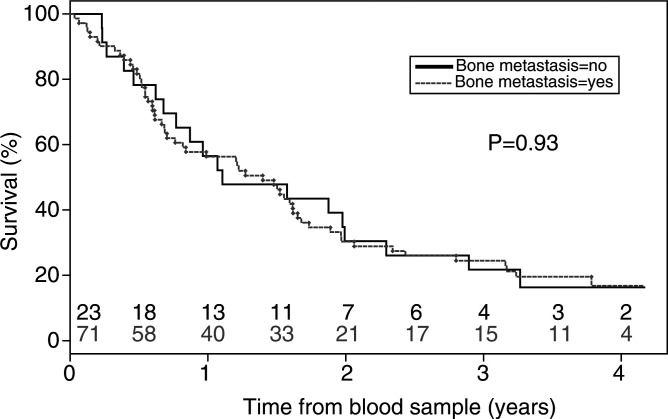
Kaplan–Meier curves for overall survival with respect to the presence of bone metastases. No prognostic value was found for the presence of bone metastases (likelihood ratio test; *P*=0.93). Patients at risk at each time point (years) are indicated for ‘bone metastasis’ (black) and ‘no bone metastasis’ (grey) patients.

The poor prognosis observed in patients with circulating tumour cells is not simply because of the more frequent presence of bone metastases: as expected, bone metastases were not a particularly negative prognostic feature for breast cancer patients with stage IV disease (Figure 4

, *P*=0.93).

## DISCUSSION

Many methods have been evaluated to reliably detect circulating tumour cells in breast cancer and in other solid malignancies, with the purpose to serve as prognostic marker. All are either based on the presence of epithelial or tumour-specific antigens in circulating cells or are based on the demonstration of RNA-species in peripheral blood cells that indicate the presence of epithelial or tumour cells. However, assays of both types have been associated with false-positive results.

Recently, the real-time PCR provided a major step forward in the power to quantitatively analyse low abundance RNA species (Bieche *et al*, 1999) and may ensure the specificity that is required for a clinically useful test. Using this method, we have recently described the identification of four marker genes for the detection of occult tumour cells in breast cancer patients and determined how the respective expression levels can be combined by QDA to allow the avoidance of false-positive results (Bosma *et al*, 2002). In the peripheral blood of a series of patients with metastatic breast cancer who were receiving systemic treatment but were otherwise unselected, 30% of the patients revealed a positive QDA. Here we show that this positive QDA value is associated with the presence of bone metastases and predicts a significantly shortened survival, whereas bone metastases do not.

Our results show that the test has a clear biological correlate and may truly represent the presence of circulating breast cancer cells. It is tempting to speculate that particularly tumour cells lodging in the bone marrow, that are responsible for bone metastases, may spill over into the blood and circulate through the body. It could also be that our patients with bone metastases and liver metastases had a larger total tumour volume than the other patients. It is not possible to determine this retrospectively. In fact, a multivariate analysis for survival that did take the site of metastasis and form of therapy into account, resulted in an increase of the *P*-value for the QDA score for survival to *P*=0.094, and was thus not significant (data not shown). Our test for circulating tumour cells was, however, not designed to predict progression-free survival, but rather to establish a biological correlate.

**Figure 5:**
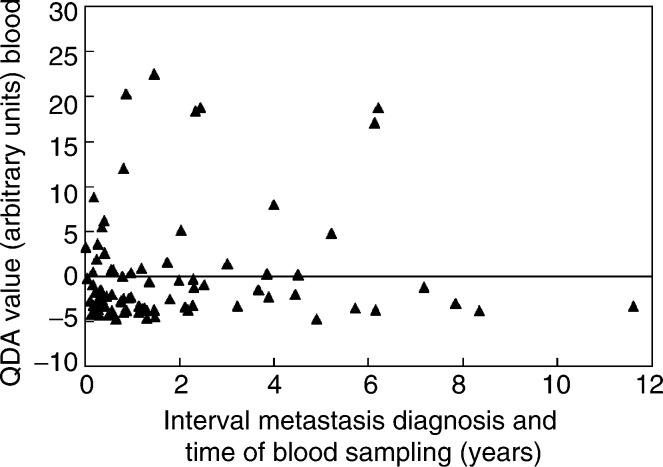
Distribution of the QDA value with respect to the interval between the detection of the first metastasis and the time of blood sampling for 86 breast cancer patients.

We could, however, exclude the possibility that the interval between the date of the first metastasis detected and the time, at which the blood sample was drawn, influenced the QDA value (Figure 5

). For 86 patients, the time of the detection of the first metastasis was available. In the interval of 6 months between the date of the first metastasis and blood sampling, 29% (eight out of 28) of the patients have a positive QDA value, compared with 29% (nine out of 31) in the subsequent interval of one-and-a-half years and 37% (10 out of 27) in the interval between 2 and 12 years. This shows that the positivity for the four marker genes is independent from the time when the blood sample is drawn in a metastasized patient. Thus, the detection of circulating tumour cells is not correlated with initial time or interval after the diagnosis of metastatic disease.

At present, it is not clear whether patients with a positive circulating tumour cell test would benefit from different, maybe more intensive treatment. The presence of micrometastatic disease and of circulating tumour cells may be of much greater importance in the management of patients with early (stages I – III) breast cancer. In this situation, prognostic information is essential to appropriately select patients for adjuvant therapy, such as hormone treatment and/or chemotherapy. We have recently shown, using expression profiling that even in early, lymph node-negative tumours, a group of patients can be identified who have a poor prognosis (van 't Veer *et al*, 2002). A reliable circulating tumour cell assay could help in the identification of additional mRNA-expression patterns that are associated with this unfavourable biologic property and could also provide independent information further narrowing down the specific risk category.
